# Optimal Design of Double-Walled Corrugated Board Packaging

**DOI:** 10.3390/ma15062149

**Published:** 2022-03-15

**Authors:** Damian Mrówczyński, Anna Knitter-Piątkowska, Tomasz Garbowski

**Affiliations:** 1 Doctoral School, Department of Biosystems Engineering, Poznan University of Life Sciences, Wojska Polskiego 28, 60-637 Poznań, Poland; damian.mrowczynski@up.poznan.pl; 2 Institute of Structural Analysis, Poznan University of Technology, Piotrowo 5, 60-965 Poznań, Poland; anna.knitter-piatkowska@put.poznan.pl; 3 Department of Biosystems Engineering, Poznan University of Life Sciences, Wojska Polskiego 50, 60-627 Poznań, Poland

**Keywords:** non-local sensitivity analysis, numerical homogenization, optimal packaging, box compressive strength, critical load, orthotropic plate, edge crush resistance, bending stiffness, corrugated cardboard

## Abstract

Designing corrugated board packaging is a real challenge, especially when the packaging material comes from multiple recycling. Recycling itself is a pro-ecological and absolutely necessary process, but the mechanical properties of materials that are processed many times deteriorate with the number of cycles. Manufacturers are trying to use unprecedented design methods to preserve the load-bearing capacity of packaging, even when the material itself is of deteriorating quality. An additional obstacle in the process of designing the structure of paper packaging is the progressive systematic reduction of the grammage (the so-called lightweight process) of corrugated cardboard. Therefore, this research presents a critical look at the process of optimal selection of corrugated cardboard for packaging structures, depending on the paper used. The study utilizes analytical, simplified formulas to estimate the strength of cardboard itself as well as the strength of packaging, which are then analyzed to determine their sensitivity to changes in cardboard components, such as the types of paper of individual layers. In the performed sensitivity analysis, numerical homogenization was used, and the influence of initial imperfections on the packaging mechanics was determined. The paper presents a simple algorithm for the optimal selection of the composition of corrugated cardboard depending on the material used and the geometry of the packaging, which allows for a more conscious production of corrugated cardboard from materials derived, e.g., from multiple recycling.

## 1. Introduction

According to the *Paper and Paperboard Packaging Market* report [1], the sector of global paper industry in 2021 was estimated to value USD 199.8 billion and is forecasted to attain USD 254.5 billion by 2026, at a Compound Annual Growth Rate (CAGR) of 5.0% during the term of reference. Such a tremendous growth is driven, among others, by a huge demand of paper packaging material in the pharmaceutical, cosmetics, food, and beverage industry. Amid the COVID-19 crisis, consumer habits have changed considerably, leading to a strong speedup of e-commerce shipments and other home delivery services, which have impacted the packaging industry by increasing the need for paper and cardboard containers.

The current environmental pollution is also a good trigger for the development of the corrugated board market in view of the fact that cellulose packaging is widely believed to be highly ecological; therefore, a shift has occurred from the use of plastic to that of paper and paperboard. Corrugated cardboard packaging companies focus also on shelf-ready packaging (SRP) or retail-ready packaging (RRP), which significantly improves unpacking and displaying products in stores. Thanks to this, brands have an excellent opportunity to shape the store space more independently and to distinguish their products with original printed packaging.

The high requirements of such a demanding market with strong competition promote manufacturers’ interest in finding innovative solutions for paper/cardboard components and the use of kraft or recycled paper and new packaging designs, e.g., by facilitating product returns or lighter fluting and allowing for a reduction in the dimensions, weight, as well as cost of the packages, provided that the boxes are of adequate load-bearing capacity. Corrugated cardboard is a layered structure whose strength is determined by the individual ply selection and combination, especially given the fact that the mechanical strength of paperboard depends on two characteristic, i.e., the in-plane directions of orthotropy—perpendicular to the main axis of the fluting and parallel to the paperboard fiber alignment machine direction (MD), as well as parallel to the fluting cross direction (CD). This paper presents a critical look at the process of optimal selection of five-layer corrugated cardboard for packaging structures, depending on the paper used, as a continuation of the discussion presented for three-ply cardboard by Mrówczyński et al. [2].

Taking into account all the above-mentioned conditions, the natural consequence of the development of the cardboard packaging market is a rapid and intensive progress in scientific research in this field. Scientists all over the world, through the years, have been proposing a great deal of methods for the estimation of cardboard load-bearing capacity. In a broad sense, one can distinguish analytical, numerical, and experimental approaches.

Analytical methods were proposed as early as in the 1950s [3,4,5] and identify paper, board, and box parameters [6] using formulae. To the first group of parameters belong the ring crush test (RCT), the Concora liner test (CLT), the liner type, the weights of liner and fluting, the corrugation ratio, and a constant related to fluting. The second one includes thickness, flexural stiffnesses in MD and CD, the results of the edge crush test (ECT), and moisture, whereas the third one comprises the dimensions and perimeter of the box, the applied load ratio, the stacking time, and the buckling and printed ratios. Fast and simple solutions for practical applications in the packaging industry can be found by employing the McKee analytical formula [5], which is commonly used but is only applicable to simple standard boxes. Therefore, this approach is still being developed, and numerous studies have focused on, e.g., the modification of constants and exponents, the expansion of the range of cutting methods and equipment [7], the introduction of the dimensions of the box [8], or the incorporation of the Poisson’s ratio [9]. A further improvement of the above approach to solve more complex problems was presented in Avilés et al. [10]. The buckling phenomena of the orthotropic cardboards were examined in Garbowski and Knitter-Piątkowska [11], and, recently, the analytical determination of the bending stiffness (BS) of a five-layer corrugated cardboard with imperfections was discussed in [12].

A recognized and valued numerical technique is the finite element method (FEM), also in terms of cardboard strength assessment. Some studies discussed the mechanical properties of cardboard during the FEM simulation of creasing [13,14,15,16,17,18], whereas others performed numerical strength estimations of corrugated board packages [19,20,21,22]. The cohesive zone method has been applied for the stress analysis in adhesively bonded joints of the corrugated sandwich structure [23] and for the prediction of the mechanical degradation of the corrugated sandwich beam [24], with verification of the results by FEM. FEM has also been employed to examine the torsional and transversal stiffness of orthotropic paper materials [25,26,27] and the bending stiffness [28,29] and buckling or post-buckling phenomena [30] of cardboard.

Finite element analysis of hot melt adhesive joints in carton board was performed by Hallbäck et al. [31]. Because of the anisotropy of paper materials and the layered structure of cardboards, carrying out numerical simulations is a challenging task, since it is necessary to know the material parameters of each layer. A remedy to this situation is a procedure called homogenization. This method consists in determining the equivalent stiffnesses and effective thicknesses of a model, which allows to reduce the layered structure to one single layer.

The equations of the classical theory of strength of materials or the classical theory of laminates lie at the root of analytical homogenization [32], whilst numerical homogenization is based on the FEM. In this approach, first, a numerical model of a representative volume element (RVE) is created [33]. The use of asymptotic homogenization was presented [34,35]. In case of a corrugated board, the homogenization may be conducted in two ways, i.e., homogenization to one layer or homogenization of fluting to the inner layer of the laminate. This procedure has been widely utilized in recent years [36,37,38,39,40,41,42,43,44,45] because of a significant saving in computation time while preserving the precision of the results.

Experimental measurements performed to determine the strength of corrugated boards are commonly performed in the paper industry. Compressive, tensile, or bursting strength tests are the main physical examinations carried out. The most common are the box compression test (BCT), the bending stiffness (BS), and the edge crush test (ECT) [12,46,47,48]. The crushing of single- and double-walled corrugated boards was discussed in Gajewski et al. [49] and Garbowski et al. [50]. Moreover, the bending test (BNT), the shear stiffness test (SST), and the torsional stiffness test (TST) are also relevant. Bursting and humidity testing are performed as well.

The method called video extensometry allows gathering data from the exterior surface of the specimen. During the testing, the relative distances between pairs of points tracked on images registered at different force values are measured [51,52]. This procedure is comparable to digital image correlation (DIC), which is a full-field non-contact optical measurement technique, yet it is simpler. A significant advantage of this method is the very high accuracy of data capture, which has made it valuable in the field of experimental mechanics [27,53,54,55,56,57,58].

Cardboard is ideal for shaping packaging material; however, one has to bear in mind that there are many factors that reduce its load-bearing capacity [59]. These include the presence of ventilation holes and perforations or indentations [60,61,62,63,64], shifted creases on flaps [65], time and conditions of storage [66,67], and the stacking load [42,68,69]. The risk of failure to meet the guaranteed load-bearing capacity cannot be disregarded. When evaluating the strength of cardboard packaging, one has to take into account the influence of box geometry and the composition as well as the arrangement of the corrugated board layers on the change of the buckling force, edge crushing (ECT), and compressive box strength resistance (BCT). Important is the fact that the behavior of cardboard strictly depends on its dimensions, i.e., for tall packaging, the buckling strength is crucial, while for low and stocky boxes, a high edge crush strength is essential.

The optimum choice of the composition of the corrugated board layers is of utmost importance for the load capacity of the packaging. The procedure described in this paper makes it possible to identify the components that affect the strength of packages of diverse dimensions and to estimate their effect. The study utilizes analytical, simplified formulas to estimate not only the strength of cardboard itself, but also the strength of packaging. These parameters are then analyzed to determine their sensitivity to changes in the cardboard components, namely, the types of paper of individual layers. In the performed sensitivity analysis, numerical homogenization and the study of the influence of initial imperfections on the packaging mechanics were carried out. The use of non-local sensitivity analysis allowed for a critical look at the process of optimal selection of corrugated cardboard for the packaging structure, depending on the paper used. A novelty that distinguishes the presented research from others is the proposed, complete algorithm for the optimal selection of the components of a five-layer corrugated cardboard depending on the material used and the geometry of the packaging.

## 2. Materials and Methods

### 2.1. Material Parameters and Corrugated Cardboard Geometry

**Figure 1:**
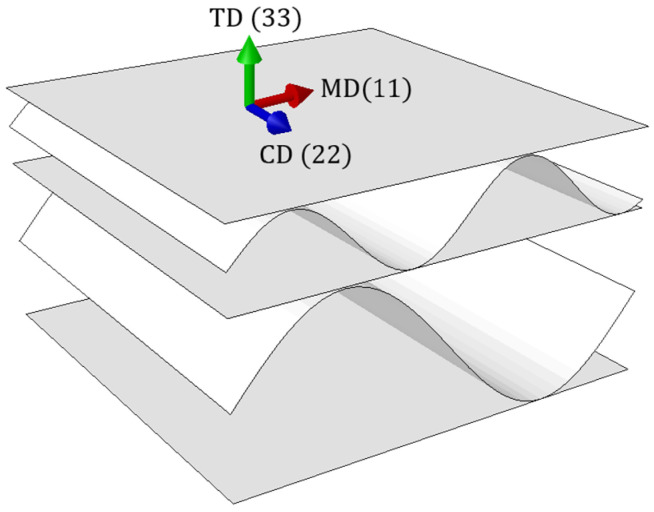
Material orientation. Machine direction (MD), cross direction (CD), and thickness direction (TD). The notation 1, 2, 3 refers to the principal orthotropic directions.

Corrugated cardboard is highly orthotropic, mainly due to the fact that it is a fibrous material. Its mechanical properties depend on the orientation of the fibers in the corrugated cardboard layers. Most fibers run along the paper web, which is called the machine direction (MD). The second main direction is the direction perpendicular to the MD, called the cross direction (CD), see Figure 1. Corrugated cardboard, because of its fiber orientation, is stiffer along the wave direction. Weaker mechanical properties in the CD are compensated by the take-up factor of the corrugated layers.

The most important mechanical properties of corrugated board are the moduli of elasticity in the machine and cross directions, $E_{1}$ and $E_{2}$, respectively, and the compressive strength in the cross direction $SCT_{CD}$. They can be calculated based on the grammage of the paper while using the MONDI specifications [70]. The method of determining the mechanical properties is shown below (see Table 1). Both moduli of elasticity can be calculated from the formulas:

(1) $$E_{1}=TS_{MD}\frac{grm}{thk},\;\;\;\;\;\;\;\;\;\;\;E_{2}=TS_{CD}\frac{grm}{thk},$$

where $TS_{MD}$ and $TS_{CD}$ are the tensile stiffness indexes in MD and CD (Nmm/g), thk is the thickness of the paper (mm), and grm is the paper grammage ($g/m^{2}$). It is assumed that the paper thickness equals to 160 μm and corresponds to a grammage of 100 $g/m^{2}$.

The remaining parameters necessary to describe the orthotropic material can be directly determined from the moduli of elasticity $E_{1}$ and $E_{2}$. The Poisson’s ratio $\nu_{12}$ and the in-plane shear stiffness $G_{12}$ can be calculated from the empirical formulas [71]:

(2) $$\nu_{12}=0.293\sqrt{\frac{E_{2}}{E_{1}}}\;,\;\;\;\;\;\;\;\;\;\;\;\;\;\;\;G_{12}=0.387\sqrt{E_{1}E_{2}}\;.$$

The transverse shear stiffnesses are approximated by the formulas [72]:

(3) $$G_{13}=\frac{E_{1}}{55}\;,\;\;\;\;\;\;\;\;\;\;\;\;\;\;\;G_{23}=\frac{E_{2}}{35}\;.$$

The geometric parameters of the waves, such as height, period, and take-up factor, are selected based on the wave type, as shown in Table 2.

### 2.2. Homogenization Technique

**Figure 2:**
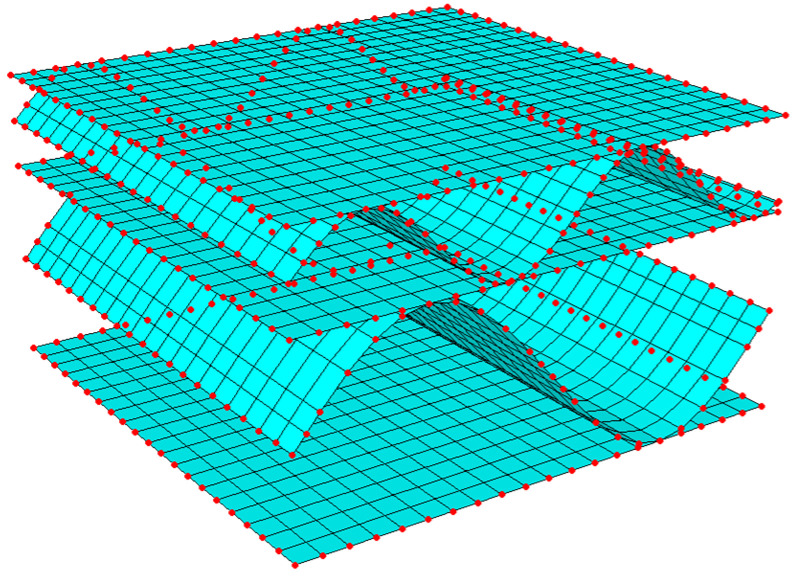
Finite elements and nodes (external, in red color) of the RVE of a double-walled corrugated board.

The full 3D model of cardboard, very burdensome in numerical calculations, can be simplified to a single layer with equivalent mechanical properties by means of numerical homogenization. In this paper, a method based on elastic energy equivalence proposed by Biancollini [33] and extended by Garbowski and Gajewski [45] is presented. This method uses a representative volume element (RVE), i.e., a small and periodic fragment of the entire corrugated cardboard structure, which is then transformed into a simplified shell model. The most important information on the applied homogenization method is presented below. The entire theoretical derivation can be found in [45]. The basic equation of the linear finite element method is as follows:

(4) $$K_{e}\;u_{e}=F_{e}\;,$$

where $K_{e}$ is a global stiffness matrix condensed to the external nodes of the RVE, $u_{e}$ is a displacement vector, and $F_{e}$ is a vector of nodal forces. The subscript e indicates values in the RVE external nodes. In Figure 2, the finite element mesh and external nodes of the RVE are presented.

In order to compute the condensed stiffness matrix, static condensation needs to be used, which removes certain unknown degrees of freedom (DOF) and only leaves the important degrees of freedom, known as primary unknowns. In the analyzed case, external nodes were left, and internal nodes were removed. The global stiffness matrix was condensed to the external nodes while applying the following formula:

(5) $$K_{e}=K_{ee}-K_{ei}\;K_{ii}{}^{-1}\;K_{ie}\;,$$

where the subarrays represent the stiffness matrices of the internal (subscript i) and external (subscript e) nodes:

(6) $$\left[\begin{array}{cc} K_{ee} & K_{ei}\\ K_{ie} & K_{ii} \end{array}\right]\left[\begin{array}{c} u_{e}\\ u_{i} \end{array}\right]=\left[\begin{array}{c} F_{e}\\ 0 \end{array}\right]\;.$$

After condensation of the model to the external nodes, the total strain energy became equal to the work of external forces on the corresponding displacements:

(7) $$E=\frac{1}{2}\;u_{e}^{T}\;F_{e}\;.$$

The presented method utilizes the principle that total energy balance between the full 3D model and the simplified shell model must be ensured. For this reason, it was necessary to appropriately determine the displacements and define the membrane and bending behavior [45]. There is a relationship between the generalized displacements and the generalized strains in the external nodes of the RVE:

(8) $$u_{i}=H_{i}\;\varepsilon_{i}\;,$$

where the $H_{i}$ matrix is defined by the coordinates of each node ($x_{i}=x$, $y_{i}=y$, $z_{i}=z$):

(9) $${\left[\begin{array}{c} u_{x}\\ u_{y}\\ u_{z}\\ \theta_{x}\\ \theta_{y} \end{array}\right]}_{i}={\left[\begin{array}{cccccccc} x & 0 & y/2 & xz & 0 & yz/2 & z/2 & 0\\ 0 & y & x/2 & 0 & yz & xz/2 & 0 & z/2\\ 0 & 0 & 0 & -x^{2}/2 & -y^{2}/2 & -xy/2 & x/2 & y/2\\ 0 & 0 & 0 & 0 & -y & -x/2 & 0 & 0\\ 0 & 0 & 0 & x & 0 & y/2 & 0 & 0 \end{array}\right]}_{i}{\left[\begin{array}{c} \varepsilon_{x}\\ \varepsilon_{y}\\ \gamma_{xy}\\ \kappa_{x}\\ \kappa_{y}\\ \kappa_{xy}\\ \gamma_{xz}\\ \gamma_{yz} \end{array}\right]}_{i}\;.$$

The transformation matrix, $H_{i}$, allows relating the generalized displacements to the generalized strains in external nodes of the RVE model. Details on the derivation of the transformation matrix can be found in [33] for the Kirchhoff–Love theory and in [45] for Reissner–Minding plates. Considering the elastic strain energy equation:

(10) $$E=\frac{1}{2}u_{e}^{T}\;K\;u_{e}=\frac{1}{2}\varepsilon_{e}^{T}\;H_{e}^{T}\;K\;H_{e}\;\varepsilon_{e}$$

and analyzing the basic load states, such as bending, tension, and transverse shear, the elastic internal energy can be determined as:

(11) $$E=\frac{1}{2}\epsilon_{e}^{T}\;H_{k}\;\varepsilon_{e}\left\{area\right\}\;.$$

The stiffness matrix for the homogenized RVE of the corrugated cardboard can be computed from:

(12) $$H_{k}=\frac{H_{e}^{T}\;K\;H_{e}}{area}\;.$$

The $H_{k}$ matrix includes the stiffness matrices **A**, **B**, **D**, and **R** as shown below:

(13) $$H_{k}=\left[\begin{array}{ccc} A_{3\times 3} & B_{3\times 3} & 0\\ B_{3\times 3} & D_{3\times 3} & 0\\ 0 & 0 & R_{2\times 2} \end{array}\right]\;,$$

where the subarray **A** represents tensile and shear stiffnesses, the subarray **B** represents the matrix linking tensile and bending stiffnesses, the subarray **D** contains bending and torsional stiffnesses, and the subarray **R** represents transverse shear stiffnesses.

The matrix **B** for symmetrical cross sections is the zero matrix. In the case of asymmetrical double-walled corrugated cardboards, which is the subject of the work, non-zero components appear in matrix **B**, which subsequently affects the values of matrix **D**. This problem can be solved by selecting a neutral axis that minimizes matrix **B**. The uncoupled matrix **D** can also be determined from the following formula:

(14) $$D=D^{\prime }-BA^{-1}B\;,$$

where D’ represents bending and torsional stiffnesses for non-zero matrix **B**.

### 2.3. Edge Crush Test (ECT)

**Figure 3:**
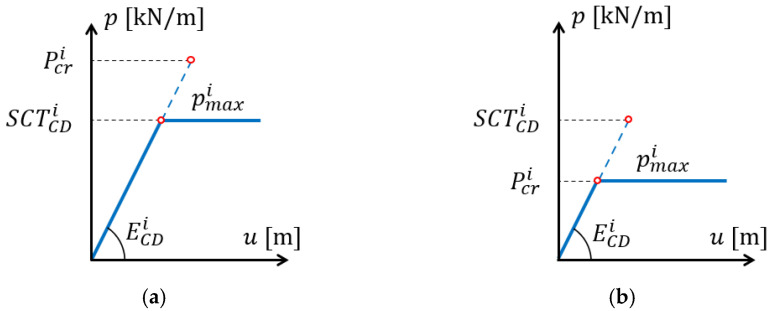
Maximum load of the i-th layer: (**a**) case where the compressive strength occurs first; (**b**) case where the critical load occurs first.

Analytically, the ECT value, used to calculate the BCT value, can be determined as the sum of the strength of all layers, taking into account the take-up factor of the waves:

(15) $$\mathrm{ECT}=\overset{n}{\underset{i=1}{\sum }}\;p_{max}^{i}\alpha_{i}\;,$$

where $p_{max}^{i}$ is the maximum load of the i-th layer, and $\alpha_{i}$ is the take-up factor (see Table 2). The maximum load of the layer can be the critical load $P_{cr}^{i}$ or the compressive strength $SCT_{CD}{}^{i}$, whichever is achieved first (see Figure 3). The maximum load is:

(16) $$p_{max}^{i}=\min\left(SCT_{CD}^{i},\;P_{cr}^{i}\right)\;.$$

The critical load can be determined in many ways, taking into account various factors. An overview of possible cases was presented by Garbowski et al. [11]. For the ECT calculations, the critical load value for rectangular orthotropic panels can be determined from a simplified formula:

(17) $$P_{cr}^{L}=\frac{1}{\alpha^{2}}\left[D_{11}\alpha^{4}+2\left(D_{12}+2D_{33}\right)\alpha^{2}\beta^{2}+D_{22}\beta^{4}\right]\;,$$

where:

(18) $$\alpha =\frac{m\pi }{H}\;,\;\;\;\;\;\;\;\;\;\;\beta =\frac{\pi }{L}\;,$$

(19) $$D_{11}=\frac{1}{w}E_{1}I\;,\;\;\;\;\;\;\;\;\;\;D_{22}=\frac{1}{w}E_{2}I\;,$$

(20) $$D_{12}=\frac{\nu_{21}}{w}E_{1}I=\frac{\nu_{12}}{w}E_{2}I\;,\;\;\;\;\;\;\;\;\;\;D_{33}=G_{12}I\;,$$

(21) $$I=\frac{t^{3}}{12}\;,\;\;\;\;\;\;\;\;\;\;w=1-\nu_{12}\nu_{21}\;,$$

where m is the number of half-waves for which $P_{cr}^{L}$ reaches the minimum, $E_{1}$ and $E_{2}$ are the moduli of elasticity in MD and CD, respectively, $\nu_{12}$ and $\nu_{21}$ are the Poisson’s coefficients in the plane, $G_{12}$ is the in-plane shear modulus, and t is the thickness of the panel.

### 2.4. Box Compression Test (BCT)

The compressive strength can be calculated using the ECT value and the critical loads of the packaging walls. For a rectangular package, the BCT value can be determined from the following formula:

(22) $$\mathrm{BCT}={\mathrm{ECT}}^{0.75}\left[\gamma_{L}{\left(P_{cr}^{L}\right)}^{0.25}L+\gamma_{B}{\left(P_{cr}^{B}\right)}^{0.25}B\right]\;,$$

where $\gamma_{L}$ and $\gamma_{B}$ are the reduction coefficients, and $P_{cr}^{L}$ and $P_{cr}^{B}$ are the critical loads of the packaging walls. The reduction coefficients can be computed from the following formulas:

(23) $$\gamma_{L}=\sqrt{\frac{L}{B}}\;,\;\;\;\;\;\gamma_{B}=1\;,\;\;\;\mathrm{if}\;L\leq B\;,\;\gamma_{L}=1\;,\;\;\;\;\;\gamma_{B}=\sqrt{\frac{B}{L}}\;,\;\;\;\mathrm{if}\;L>B\;.\;$$

In the case of a relatively thick corrugated cardboard (high waves or double-walled cardboards) and low transverse shear modulus (due to unintentional crushing or the lamination process), it is crucial to take into account the transverse shear stiffness in the calculation of the critical loads of the packaging walls. The transverse shear stiffness is included in the equation:

(24) $$P_{cr}^{L}=\frac{1}{\alpha^{2}}\frac{M}{N}\;,$$

where:

(25) $$M=D_{11}\alpha^{4}+2\left(D_{12}+2D_{33}\right)\alpha^{2}\beta^{2}+D_{22}\beta^{4}+\left(\frac{\alpha^{2}}{R_{44}}+\frac{\beta^{2}}{R_{55}}\right)c_{1}\;,$$

(26) $$N=1+\frac{c_{1}}{R_{44}R_{55}}+\frac{c_{2}}{R_{55}}+\frac{c_{3}}{R_{44}}\;,$$

(27) $$c_{1}=c_{2}c_{3}-c_{4}{}^{2}>0\;,$$

(28) $$c_{2}=D_{11}\alpha^{2}+D_{33}\beta^{2}\;,$$

(29) $$c_{3}=D_{33}\alpha^{2}+D_{22}\beta^{2}\;,$$

(30) $$c_{4}=\left(D_{12}+D_{33}\right)\alpha \beta \;.$$

### 2.5. Bending Stiffness with Imperfections

In the above buckling formulas (Equations (25)–(30)), the bending stiffnesses $D_{11}$ and $D_{22}$ are present, which in the case of a corrugated board with an unsymmetrical cross section, may slightly differ depending on the bending direction (sign of the bending moment). This case was analyzed and presented by Garbowski and Knitter-Piątkowska [12] for a five-play corrugated board bent in MD (i.e., $D_{11}$ stiffness). The main reason for the disparities is the different number of compressed flat layers, which, in the presence of even very small imperfections, cause noticeable discrepancies in bending stiffness. The bending stiffness in MD can be computed from:

(31) $$D_{11}=\overset{N}{\underset{i=1}{\sum }}\frac{E_{1,i}t_{i}\delta_{i}}{1+6f_{i}^{2}t_{i}^{-2}}\frac{z_{i}}{\phi }\;,$$

where $E_{1,i}$ is the Young’s modulus in MD of i-th liner, $t_{i}$ is thickness of i-th liner, $\delta_{i}$ is the axial deformation of i-th liner, $z_{i}$ is the distance from the i-th liner to the neutral axis of the entire cross section, $f_{i}$ is an initial imperfection of the i-th liner, ϕ is the rotation of the cross section. It is worth noting that in the case of bending of asymmetrical sections, the value of $f_{i}$ is different from zero only for the liners that are in compression. In a similar way, one can derive the stiffness in CD, i.e., $D_{22}$:

(32) $$D_{22}=\overset{N}{\underset{i=1}{\sum }}\frac{E_{2,i}t_{i}\delta_{i}}{1+3f_{i}^{2}t_{i}^{-2}}\frac{z_{i}}{\phi }\;.$$

Using the above formulations, two different values of bending stiffness, in both the MD and the CD directions, are obtained. Therefore, both cases should be taken into account when calculating the critical load in Equation (24), depending on the direction of the initial imperfections of the entire corrugated board.

### 2.6. Non-Local Sensitivity Analysis

The non-local sensitivity of corrugated cardboard was tested for edgewise crush resistance (ECT), box compressive strength (BCT), and critical loads of packaging walls ($P_{cr}$). The model parameters were the grammage of the corrugated cardboard layers or the bending stiffnesses $D_{11}$ i $D_{22}$, which were placed in the vector x. The sensitivity at a specific point in the parameter space can be calculated by determining a numerical gradient while using, e.g., the central difference, according to the formula:

(33) $$s=\frac{h\left(x+e_{i}\Delta x_{i}\right)-h\left(x-e_{i}\Delta x_{i}\right)}{2\Delta x_{i}}\frac{x_{i}}{h\left(x\right)}\;$$

where h(x) is the quantity for which the sensitivity is determined (ECT, BCT or $P_{cr}$), $\Delta x_{i}$ is a small perturbation of the *i*-th parameter, $h\left(x-e_{i}\Delta x_{i}\right)$ is the change of the determined quantity, and $e_{i}$ is the unit vector of the *i*-th parameter in the vector x.

**Figure 4:**
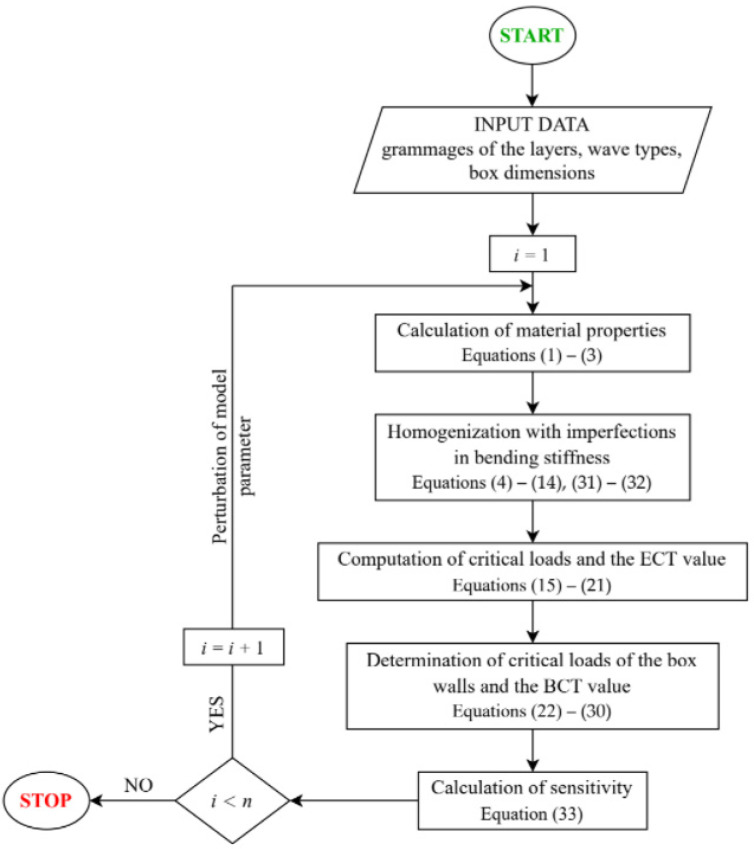
Flowchart of the algorithm for the determination of the non-local sensitivities of a five-play corrugated board.

In the above description, the term ’non-local‘ means that information about the gradients of the studied quantities is collected in the full range of the analyzed parameter at many points in the parameter space, not only locally at one point in this space. Figure 4 shows an algorithm for the determination of the non-local sensitivity. In the flowchart, i is the number of iterations, and n is the total number of the perturbing parameters.

## 3. Results

The most effective tool for determining the optimal layer configuration of a multi-ply corrugated board for packaging is the non-local sensitivity analysis. With its help, it is possible to check which layers (e.g., what basis weight, what type of paper) work best and in which packaging structures. All sensitivity values presented below were computed from Equation (33), where the variable h indicated ECT, $P_{cr}$, or BCT values. First, we analyzed the sensitivity of ECT, the value of which was calculated from Equation (15). The ECT value is influenced by the stiffnesses in the machine (MD) and cross (CD) directions and by the compressive strength in the CD (through the critical load). It follows that the ECT value depends on the grammage of the cardboard layers, see Equation (1).

Four double-walled corrugated cardboards (BC, EB, EC, EE) for different combinations of layer grammage were analyzed. The combinations were based on liners grammage from 100 every 20 to 200 $g/m^{2}$ and fluting grammage from 80 every 20 to 160 $g/m^{2}$, which gave in total 5400 combinations for each cardboard.

**Figure 5:**
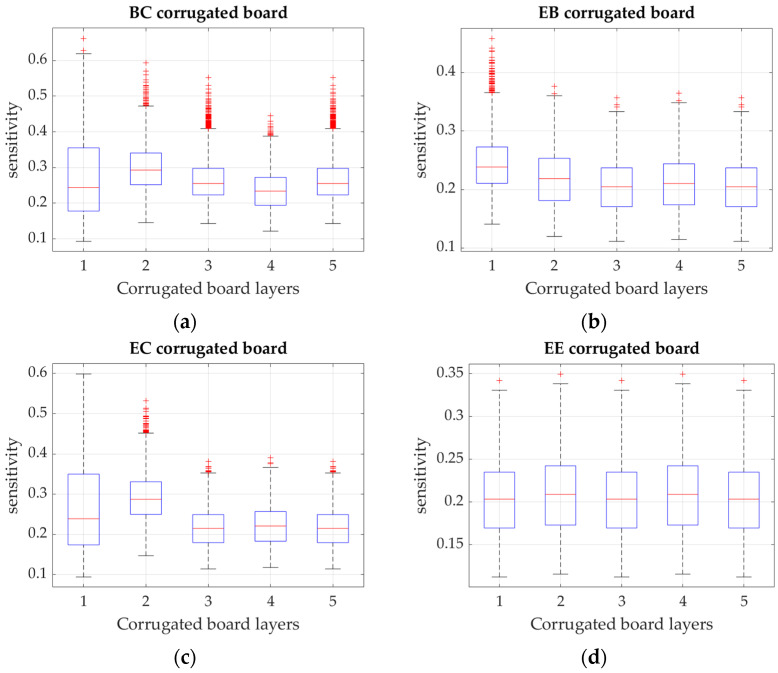
Sensitivity of ECT in relation to the layer grammage perturbation (1—bottom liner, 2—higher flute, 3—middle liner, 4—lower flute, 5—upper liner) for: (**a**) BC, (**b**) EB, (**c**) EC, and (**d**) EE corrugated cardboard.

In Figure 5, the sensitivity of ECT to the change in grammage of the cardboard layers is presented. The median values are marked by red horizontal lines, and the bottom and top blue edges of the box indicate the 25th and 75th percentiles, respectively. The whiskers extend to the most extreme data points not considered outliers, and the outliers are plotted individually using the ‘+’ marker symbol.

**Figure 6:**
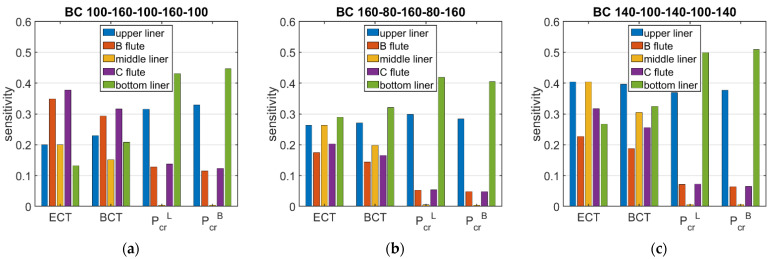
Average sensitivity of ECT, $P_{cr}$, and BCT of all boxes in relation to the layer grammage perturbation for BC cardboard of selected grades: (**a**) 100-160-100-160-100, (**b**) 160-80-160-80-160, and (**c**) 140-100-140-100-140.

**Figure 7:**
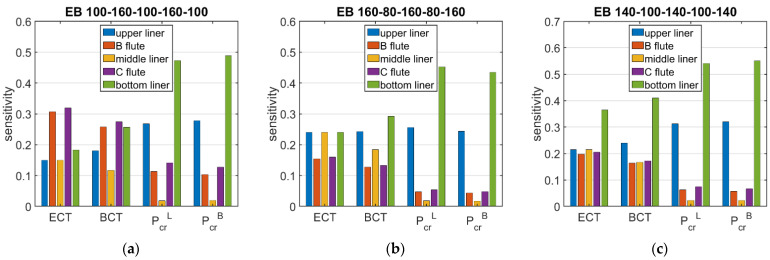
Average sensitivity of ECT, $P_{cr}$, and BCT of all boxes in relation to the layer grammage perturbation for EB cardboard of selected grades: (**a**) 100-160-100-160-100, (**b**) 160-80-160-80-160, and (**c**) 140-100-140-100-140.

Figure 6 and Figure 7 show the sensitivities of ECT, $P_{cr}$, and BCT with respect to the grammage of the cardboard layers. The presented results are average values that were obtained from 120 boxes of various dimensions. The dimensions of the box base were from 100 × 100 mm to 500 × 300 mm, and the height of the box was in the range from 50 to 500 mm.

The presented sensitivities are limited to three indices of corrugated cardboard—double-walled with a liner grammage of 100 $g/m^{2}$ and a fluting grammage of 160 $g/m^{2}$—marked as 100-160-100-160-100, 160-80-160-80-160, and 140-100-140-100-140. The results are presented for the BC cardboard (see Figure 6) and the EB cardboard (see Figure 7).

Table 3 and Table 4 show the sensitivity of BCT and $P_{cr}$ depending on the bending stiffnesses for the BC and EB corrugated cardboards, respectively. The values in columns 3–5 are the average sensitivities computed for 36 boxes lower than 150 mm. The sensitivities in columns 6–8 are the average values obtained for nine packages higher than 400 mm, with a base dimension lower than 200 mm.

**Figure 8:**
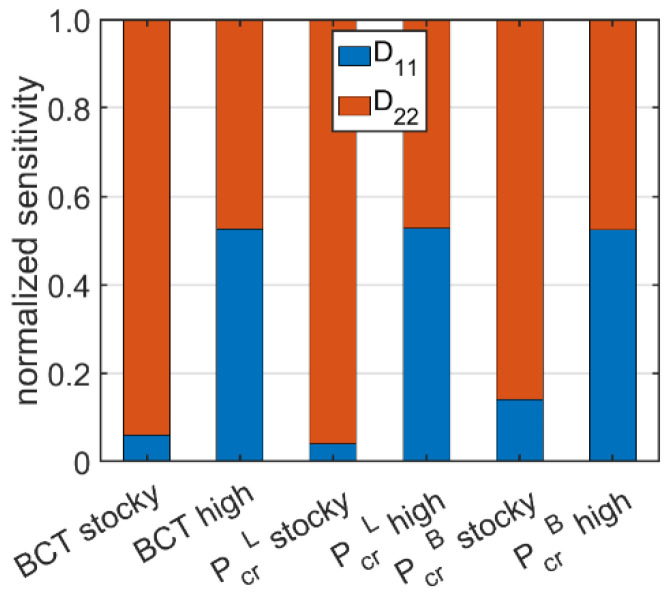
Normalized sensitivity of BCT and $P_{cr}$ regarding the $D_{11}$ and $D_{22}$ perturbation of the BC corrugated cardboard with a grade of 100-160-100-160-100.

Figure 8 presents the participation of the bending stiffnesses $D_{11}$ and $D_{22}$ in the sensitivity of BCT and $P_{cr}$. The results are shown for the BC corrugated cardboard with a grade of 100-160-100-160-100. The proportions for the remaining cases are similar; therefore, the results are shown only for cardboard of one grade.

The above-presented results summarize the sensitivities calculated according to the scheme presented in Figure 4. Most analyses were based on simplified analytical formulas; only the part regarding the numerical homogenization used the basics of the finite element method. The homogenization method has already been extensively described in [45,49,50,61].

## 4. Discussion

This study is a continuation of the work recently presented by Mrówczyński et al. [2]. What distinguishes this work from the previous one is the attention to the effects related to imperfections in the asymmetrical cross section of a five-layer corrugated cardboard. Taking into account these effects and the other specificities of a five-layer corrugated cardboard allows for drawing slightly different, but still very important, conclusions. To the best of the authors’ knowledge, there are no other scientific papers in the literature that describe in this way the influence of the composition of a corrugated board on all mechanical parameters of the structure of paper-based packaging. Therefore, the following discussion is limited to a summary of the observations of the results of this work.

The performed analyses allowed determining the sensitivity of ECT, $P_{cr}$, and BCT to small perturbations of the layer grammage and bending stiffnesses $D_{11}$ and $D_{22}$. In Figure 5, the results of the ECT sensitivity for the four analyzed corrugated cardboards (BC, EB, EC, and EE) are presented. For asymmetrical cardboards, the grammage liner with a higher wave had a more important effect than the grammage of other liners (for BC cardboard, the sensitivity was similar). All cardboards with two different waves (flutes), were more sensitive to small changes in the grammage of the higher wave than to changes in the grammage of the lower wave. For the symmetrical EE cardboard, the sensitivity of liners and flutes was similar.

Figure 6 and Figure 7 show the average sensitivities regarding the layer grammage perturbation for BC and EB cardboards, respectively. Based on of the presented results, it can be concluded that the sensitivities of the critical load of the longer and shorter walls of the box were very similar. The maximum difference between the average sensitivities was about 13%. It can be clearly seen that the value of the critical load was mostly influenced by the grammage of the external liners (from 2.44% to 5.51% when the liner grammage was changed by 10%), the fluting grammage had little influence (from 0.43% to 1.41% when changing the fluting grammage by 10%), and the influence of the middle liner grammage was negligible (from 0.04% to 0.22%).

Despite the low impact of the middle liner and fluting on the critical load of the walls, they contribute to the packaging load capacity through the ECT value, which is also a main component of the box strength (BCT). For corrugated cardboard with thick fluting (grade 100-160-100-160-100), both ECT and BCT were more sensitive to fluting perturbation than to liner perturbation. In the remaining cases (grades 160-80-160-80-160 and 140-100-140-100-140), the liners had a greater influence on the ECT and BCT values. In all cases, the external liners had a much greater impact on the load capacity than the middle liner (7% to 146%).

Table 3 and Table 4 show the sensitivity of critical load and BCT to changes in the bending stiffness. It is easy to notice that the stiffness $D_{11}$ is less important for stocky boxes than for slender ones. The change in BCT resulted from a change of $D_{11}$ by 10% was about 0.14% for stocky boxes and about 0.64% for tall boxes. The opposite trend was observed for the stiffness $D_{22}$; in fact, the change in BCT caused by a 10% change of $D_{22}$ for low boxes was about 1.79%, while that for high boxes was about 0.65%. The same relationship was seen when analyzing the sensitivity of the critical load. Comparing the sensitivities to perturbations of both stiffnesses, it can be seen that for low boxes, the bending stiffness $D_{22}$ is much more important than $D_{11}$, whereas for high boxes, both stiffnesses have a similar effect on the value of the load capacity and critical loads (see Figure 8).

## 5. Conclusions

Nowadays it is extremely important that materials are used in the best possible way in the production of various structures, including corrugated cardboard. Understanding and analyzing the impact of various factors on the load capacity of packaging is of key importance in the optimization process of both material consumption and selection of cardboard quality for a specific packaging structure. This paper presented the results obtained from numerical analyses of five-layer cardboard aimed at determining the sensitivity of certain values (indexes used to determine strength), such as edge crush resistance (ECT), critical load of the packaging walls ($P_{cr}$), and packaging load capacity (BCT), to changes in the grammage of the corrugated cardboard layers and bending stiffness $D_{11}$ and $D_{22}$. Based on the performed numerical analyses and the presented calculation results, several conclusions were formulated, that will contribute to a more optimal design of double-walled corrugated cardboard packaging and thus improve the sustainable management of natural resources.

## Figures and Tables

**Table 1:** MONDI technical data for kraftliner paper.

Property	Unit of Measure	Grammage (g/m^2^)								
		100	110	125	135	150	160	170	186	200
$SCT_{CD}$	N/mm	2.0	2.3	2.5	2.7	3.0	3.2	3.4	3.7	4.0
${\mathrm{Tensile}\;\mathrm{stiffness}\;\mathrm{index}\;}_{MD}$	Nmm/g	11					12			
${\mathrm{Tensile}\;\mathrm{stiffness}\;\mathrm{index}\;}_{CD}$	Nmm/g	5								

**Table 2:** Geometric parameters of waves.

Wave (Flute)	Wave Length (mm)	Height (mm)	Take-Up Factor (-)
B	6.5	2.46	1.32
C	8.0	3.61	1.43
E	3.5	1.15	1.27

**Table 3:** Sensitivity of BCT and $P_{cr}$ regarding the $D_{11}$ and $D_{22}$ perturbation of a BC corrugated cardboard.

Grade	Perturbed Parameter	Stocky Boxes			High Boxes		
		BCT	$P_{cr}^{L}$	$P_{cr}^{B}$	BCT	$P_{cr}^{L}$	$P_{cr}^{B}$
100-160-100-160-100	$D_{11}$	0.012	0.032	0.093	0.067	0.267	0.264
	$D_{22}$	0.183	0.775	0.571	0.060	0.239	0.240
160-80-160-80-160	$D_{11}$	0.015	0.041	0.116	0.062	0.248	0.245
	$D_{22}$	0.177	0.754	0.539	0.068	0.270	0.271
140-100-140-100-140	$D_{11}$	0.014	0.039	0.111	0.059	0.237	0.234
	$D_{22}$	0.178	0.759	0.547	0.070	0.278	0.280

**Table 4:** Sensitivity of BCT and $P_{cr}$ regarding the $D_{11}$ and $D_{22}$ perturbation of an EB corrugated cardboard.

Grade	Perturbed Parameter	Stocky Boxes			High Boxes		
		BCT	$P_{cr}^{L}$	$P_{cr}^{B}$	BCT	$P_{cr}^{L}$	$P_{cr}^{B}$
100-160-100-160-100	$D_{11}$	0.012	0.034	0.098	0.069	0.277	0.274
	$D_{22}$	0.181	0.770	0.563	0.058	0.231	0.232
160-80-160-80-160	$D_{11}$	0.015	0.042	0.119	0.064	0.254	0.251
	$D_{22}$	0.176	0.752	0.535	0.066	0.265	0.266
140-100-140-100-140	$D_{11}$	0.014	0.040	0.114	0.061	0.244	0.241
	$D_{22}$	0.177	0.756	0.542	0.068	0.272	0.274

## Data Availability

The data presented in this study are available on request from the corresponding author.
